# Acute cardiorenal decompensation in the setting of chronic renal artery stenosis treated with successful renal artery stenting. A case report

**DOI:** 10.47487/apcyccv.v6i4.515

**Published:** 2025-12-29

**Authors:** Nehaal Ahmed, Jose Arriola-Montenegro, Saad Rashid, Ian R McPhail, Benjamin Bizer, Iasmina M Craici, Samy M Riad

**Affiliations:** 1 Division of Internal Medicine, Mayo Clinic, Rochester, Minnesota, USA. Mayo Clinic College of Medicine Division of Internal Medicine Mayo Clinic Rochester, Minnesota USA; 2 Department of Nephrology and Hypertension, Mayo Clinic, Rochester, Minnesota, USA. Mayo Clinic College of Medicine Department of Nephrology and Hypertension Mayo Clinic Rochester, Minnesota USA; 3 St. George’s University College of Medicine, True Blue, Grenada. St. George’s University College of Medicine True Blue Grenada; 4 Department of Cardiovascular Medicine, Mayo Clinic, Rochester, Minnesota, USA. Mayo Clinic College of Medicine Department of Cardiovascular Medicine Mayo Clinic Rochester, Minnesota USA

**Keywords:** Renal Artery Obstruction, Hypertension, Pulmonary Edema, Acute Kidney Injury, Angioplasty, Balloon, Obstrucción de la Arteria Renal, Hipertensión Renal, Edema Pulmonar, Lesión Renal Aguda, Angioplastia de Balón

## Abstract

Atherosclerotic renal artery stenosis may present with resistant hypertension, hypertensive crisis, ischemic nephropathy, and cardiac destabilization. Although early studies of renal artery stenting showed promise, later trials found no benefit over medical therapy-yet often excluded high-risk, acute cases. We describe an 85-year-old male with cardiovascular risk factors who developed a hypertensive crisis, acute kidney injury, and flash pulmonary edema. An angiogram revealed severe ostial right renal artery stenosis. Stenting resulted in the rapid resolution of the acute crisis and restoration of baseline renal function. This case underscores the potential benefit of revascularization in select high-risk presentations of renal artery stenosis.

## RESUMEN

La estenosis aterosclerótica de la arteria renal puede manifestarse con hipertensión resistente, crisis hipertensiva, nefropatía isquémica y falla cardíaca. Aunque los estudios iniciales sobre la colocación de stents en la arteria renal mostraron resultados prometedores, ensayos posteriores no evidenciaron beneficios frente al tratamiento médico; sin embargo, con frecuencia excluyeron a pacientes agudos de alto riesgo. Presentamos el caso de un varón de 85 años con factores de riesgo cardiovascular que desarrolló crisis hipertensiva, lesión renal aguda y edema pulmonar súbito. La ecografía reveló una estenosis ostial grave de la arteria renal derecha. El implante de un stent permitió la rápida resolución del cuadro agudo y la recuperación de la función renal basal. Este caso resalta el beneficio potencial de la revascularización en el escenario de estenosis de la arteria renal de alto riesgo.

## Introduction

Atherosclerosis is the most common cause of chronic renal artery stenosis (RAS). ^1^ Although RAS can contribute to resistant hypertension (HTN) and progressive chronic kidney disease (CKD), it is frequently asymptomatic and identified incidentally. In such cases, medical management remains the mainstay of therapy, including lipid-lowering agents, antihypertensive regimens, aspirin, smoking cessation, and optimization of glycemic control. ^1^^-^^3^ However, chronic RAS may progress and manifest with high-risk features, such as progressive acute kidney injury (AKI), hypertensive emergencies, or flash pulmonary edema. In these situations, percutaneous transluminal renal artery stenting (PTRAS) has been shown to provide significant clinical benefit. ^1^^-^^4^

Here, we present the case of a patient with chronic RAS complicated by a hypertensive emergency, progressive AKI on CKD, and flash pulmonary edema, who showed marked clinical and renal recovery following PTRAS.

## Case report

An 85-year-old male was admitted with progressive abdominal pain, diarrhea, and chest pressure. His past medical history was notable for a prior 45 pack-year smoking history, coronary artery disease (CAD) with three-vessel coronary artery bypass surgery, diabetes mellitus, HTN, CKD with baseline creatinine of 1.6-1.9 mg/dL, and an estimated glomerular filtration rate (eGFR) of 37 mL/min/1.73 m2, calculated using the 2021 CKD-EPI creatinine equation. An ultrasound performed 5 years prior showed moderate right renal artery stenosis with a peak systolic velocity (PSV) of 168 cm/s.

On admission, he was afebrile, tachycardic with a heart rate of 126 beats/min, and hypertensive with a blood pressure of 247/120 mmHg. Laboratory results revealed elevated eosinophils at 3.4 x 109 and creatinine at 2.2 mg/dL. Initial troponin T was 314 ng/L (reference range < 15 ng/L), with a six-hour repeat further elevated at 388 ng/L. An EKG noted left atrial enlargement, incomplete right bundle branch block, and new ST depressions in leads V5-6. Given the chest pressure, EKG abnormalities, and elevated troponin, acute coronary syndrome and aortic dissection were considered but subsequently ruled out through cardiology evaluation and imaging. Computed tomography (CT) of the abdomen and pelvis without contrast noted extensive calcific atherosclerosis of the aorta with heavy calcification filling most of the lumen of the proximal renal arteries.

After initial treatment with intravenous labetalol 20 mg and hydralazine 20 mg every 4 hours, his blood pressure improved to 129/54 mmHg, and chest pressure resolved. While his diarrhea improved, serum creatinine increased to 4.1 mg/dL, and blood pressure increased to 188/81 mmHg despite uptitration of home antihypertensives. An echocardiogram demonstrated normal left ventricular size, generalized hypokinesis, a left ventricular ejection fraction of 37%, an estimated right ventricular systolic pressure of 51 mmHg, and an enlarged inferior vena cava with normal inspiratory collapse.

**Figure 1:**
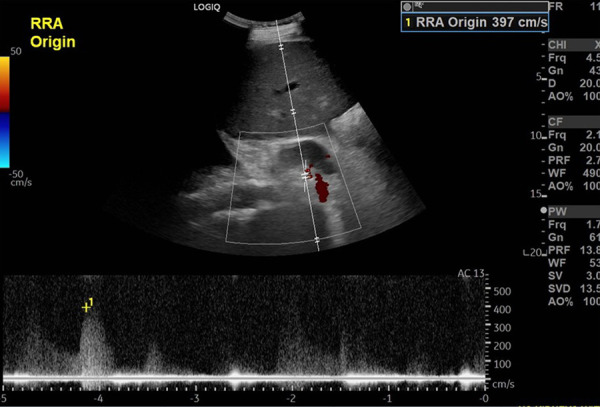
Renal ultrasound with Doppler demonstrating significant right renal artery stenosis with markedly elevated right renal artery peak systolic velocity of 397 centimeters per second (prior baseline 168 centimeters per second).

An ultrasound with Doppler revealed an atrophic left kidney with a poorly visualized left renal artery and a normal-appearing right kidney with new severe right renal artery stenosis, with a peak systolic velocity of 397 cm/s (baseline 168 cm/s) (Figure 1). The patient subsequently developed acute decompensated heart failure and required transfer to the intensive care unit for bilevel positive airway pressure with continuous intravenous clevidipine and furosemide infusion.

**Figure 2:**
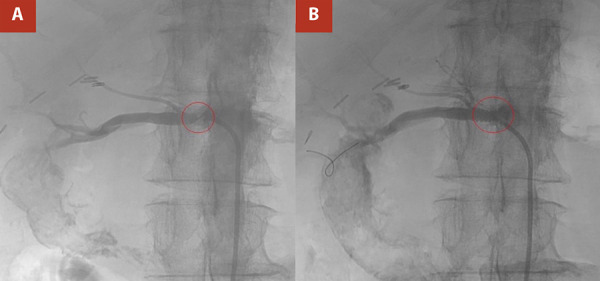
A. Fluoroscopy-guided renal angiogram demonstrating critical atherosclerotic stenosis of the ostial right renal artery. B. After deployment of a stent, the stenosis is resolved, and the right renal artery is widely patent.

Renal angiography was performed via right common femoral artery access under ultrasound guidance and local anesthesia. After successful puncture and sheath placement, severe atheromatous disease of the right iliac segment was traversed, and the abdominal aorta was cannulated. A selective right renal arteriogram demonstrated a critical, heavily calcified ostial stenosis. Following systemic anticoagulation with intravenous heparin, the lesion was crossed with a 0.014” wire. Balloon angioplasty was attempted, but residual severe narrowing persisted despite sequential dilation (Figure 2A). A 5 x 15 mm Herculink stent was then deployed and expanded to 5.5 mm, resulting in an angiographically satisfactory outcome with a widely patent lumen, brisk flow, and no distal embolization (Figure 2B).

Subsequent selective injection of the left renal artery revealed a critical, heavily calcified ostial stenosis associated with an atrophic kidney. Multiple attempts to cross the lesion were unsuccessful, and given the advanced parenchymal atrophy, no further intervention was pursued. Hemostasis was achieved with an Angio-Seal device, and the patient tolerated the procedure without complications.

**Figure 3:**
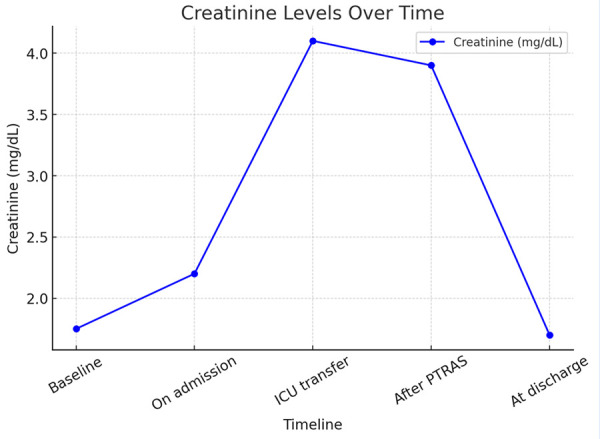
The trend of creatinine over time is depicted above. The baseline level was 1.6-9 mg/dL, with an increase to 2.2 mg/dL on admission. At the time of ICU transfer, creatinine peaked at 4.1 mg/dL. Immediately after percutaneous right renal artery stenting, creatinine remained stable at 3.9 mg/dL. However, it subsequently quickly downtrended to baseline at discharge and was 1.5 mg/dL at short-term outpatient follow-up.

Post-procedure, the patient was initiated on dual antiplatelet therapy with aspirin 81 mg and clopidogrel 75 mg daily. Over the following days, his HTN, heart failure, and AKI resolved. At outpatient follow-up, his creatinine returned to baseline at 1.5 mg/dL (Figure 3).

## Discussion

Acute decompensation in chronic RAS is rare but serious, requiring prompt recognition and intervention. Atherosclerotic RAS (ARAS) accounts for more than 90% of cases and typically affects the ostium or proximal renal artery in elderly patients with cardiovascular risk factors such as CAD, peripheral artery disease, CKD, HTN, and diabetes. ^1^^,^^3^^,^^5^^,^^6^ Chronic RAS is often asymptomatic and discovered incidentally. It may be suspected when serum creatinine rises after the initiation of angiotensin-converting enzyme inhibitors (ACEIs) or angiotensin receptor blockers (ARBs), in cases of resistant or sudden-onset hypertension, progressive CKD, or recurrent heart failure. ^1^^,^^5^ Acute RAS is less common, usually resulting from embolism, dissection, or plaque rupture. ^7^^)^ Symptoms may range from flank pain, AKI, and flash pulmonary edema to nonspecific findings such as fever or hematuria, often delaying diagnosis. ^7^^,^^8^

Renal Doppler ultrasound is a useful noninvasive modality that provides information on stenosis severity, flow velocity, and parenchymal changes, while avoiding the contrast exposure required for computed tomographic angiography (CTA) or magnetic resonance angiography (MRA). ^1^ Although noninvasive tests can strongly suggest significant RAS, invasive angiography with pressure gradient remains the gold standard; a translesional gradient of ≥20 mmHg indicates significant hypoperfusion. ^1^^,^^5^

Most ARAS patients are treated medically with statins, antihypertensives, aspirin, smoking cessation, and diabetes control. ^1^^,^^2^ While surgical revascularization exists, PTRAS is generally preferred. ^1^ Balloon angioplasty without stenting, effective for fibromuscular dysplasia, is insufficient for ARAS due to the risk of restenosis. ^1^ Randomized trials and meta-analyses comparing PTRAS to medical therapy have not shown consistent benefits in unselected, low-risk patients and highlight potential procedural risks. ^1^^,^^2^^,^^9^^-^^14^ These studies had significant limitations, including the inclusion of patients with mild to moderate RAS, those with advanced irreversible nephropathy or unilateral disease, exclusion of high-risk patients with acute presentations, lack of invasive hemodynamic assessment, and limited statistical power. ^1^^,^^3^^,^^9^^-^^14^

Severe RAS alone does not predict benefit from PTRAS; associated clinical syndromes are critical. Flash pulmonary edema remains the strongest indication, with reported mortality reduction. ^1^^,^^2^^,^^4^ Other accepted indications include refractory hypertension, oliguric AKI, or recurrent heart failure attributed to RAS. ^1^^,^^2^^,^^4^^,^^5^^,^^15^^,^^16^ The greatest benefit is seen in patients without proteinuria, advanced CKD, or ischemic fibrosis. ^3^^,^^8^ A post-hoc analysis of the CORAL trial demonstrated improved outcomes in patients with minimal proteinuria (<22.5 mg/g). ^17^ PTRAS may even reverse dialysis dependence in select patients with anuric AKI, particularly those with a solitary functioning kidney and low proteinuria. ^16^ In contrast, PTRAS is unlikely to help in advanced CKD with proteinuria >1g/day, resistive index >0.8, kidney length <8 cm, cortical thickness <0.5 cm, or prolonged dialysis dependence. ^1^^,^^3^^,^^8^

In our case, AKI was multifactorial. Prolonged diarrhea triggered hypovolemia, reducing effective intravascular volume and renal perfusion. This prerenal insult was particularly significant given the patient’s severe right renal artery stenosis and atrophic left kidney, rendering him functionally uninephric. In addition, marked eosinophilia raised concern for renal atheroembolism, possibly precipitated by hemodynamic instability or prior arterial manipulation. Superimposed on this, the patient’s hypertensive emergency reflected activation of the renin-angiotensin-aldosterone system (RAAS) in response to renal hypoperfusion. Severe hypertension not only worsened renal injury directly, but also precipitated acute cardiac dysfunction with pulmonary edema. The resulting renal venous congestion and possible low cardiac output further aggravated kidney injury, exemplifying the bidirectional interaction of the heart and kidney in the setting of RAS. The temporal resolution of renal dysfunction and hemodynamic instability following stenting strongly suggests that revascularization played a causal role in reversing this downward spiral. Importantly, minimal parenchymal disease in the right kidney and the absence of proteinuria favored a successful outcome after PTRAS. ^1^^,^^17^

Although complication rates with PTRAS are generally low, potential risks include atheroembolism, dissection, and arterial perforation, particularly in patients with advanced atherosclerosis. ^1^^,^^3^ In our patient, peripheral eosinophilia raised concern for atheroembolic kidney injury, highlighting that even when PTRAS is ultimately successful, atheroembolism can complicate the clinical course. This underscores the need for careful patient selection and emphasizes that decisions regarding renal revascularization are best made within a multidisciplinary team, balancing procedural risks against potential benefits.

This case underscores the importance of recognizing multifactorial contributors to AKI in chronic RAS, the interplay between hypertensive crisis, cardiac dysfunction, and renal congestion, and the role of timely PTRAS in high-risk patients presenting with acute heart failure or rapidly progressive renal dysfunction. In particular, revascularization was temporally associated with sustained improvement in renal function and hemodynamic stabilization, supporting a causal relationship in this high-risk setting.
